# Prevalence and determinants of multimorbidity in older Chinese adults: a nationwide cross-sectional study using CLASS data

**DOI:** 10.1186/s12877-026-07893-x

**Published:** 2026-07-06

**Authors:** Yi Li, Chenxi Zhao, Xinhao Zhuang, Songbo Jia, Xuelin Zhang, Liming Yan, Huaguang Zheng, Yiqun Pang, Bing Zhan, Xiaotian Li, Jinxi Zhang, John S. Ji, Yanjun Guo, Bingxiang Xu, Zhixiong Zhou, Qingzhu Yang

**Affiliations:** 1 Capital University of Physical Education and Sports, Beijing, 100191 China; 2 School of Sciences, Hebei University of Technology, Tianjin, 300401 China; 3 Key Laboratory of Hebei Province for Molecular Biophysics, Institute of Biophysics, School of Health Science & Biomedical Engineering, Hebei University of Technology, Tianjin, 300130 China; 4 School of Medicine, Tsinghua University, Beijing, 100084 China; 5 Beijing Tiantan Hospital, Capital Medical University, Beijing, China; 6 School of Physical Education, Southwest Petroleum University, Chengdu, Sichuan Province 610000 China; 7 Vanke School of Public Health, Tsinghua University, Beijing, 100084 China; 8 School of Public Health, Tongji Medical College, Huazhong University of Science and Technology, Wuhan, Hubei Province 430030 China

**Keywords:** Multimorbidity, Prevalence, Associated factors, Medical resources, Chinese older adults

## Abstract

**Background:**

Multimorbidity, the coexistence of two or more chronic conditions, is rising in China’s aging population, with limited data on prevalence and regional drivers.

**Methods:**

Using 2020 China Longitudinal Aging Social Survey data for adults aged ≥ 60, supplemented by official regional statistics, we defined multimorbidity from 22 self-reported conditions. A Generalized Linear Mixed Model (GLMM) with village/community-level random effects was used to identify individual-level correlates of multimorbidity, while Random Forests (RF) evaluated county-level determinants.

**Results:**

Among 11,372 participants (mean [Standard Deviation, SD] 71.6 [6.6] years), 46.03% had multimorbidity. Higher odds of multimorbidity were associated with older age (Odds Ratio [OR] = 2.24; 95% Confidence Interval [CI] 1.81–2.76), female (OR = 1.32; 95% CI 1.20–1.45), receiving ≥ 3 social security benefits (OR = 1.64; 95% CI 1.09–2.48), and obesity (OR = 1.90; 95% CI 1.48–2.44). Lower odds were associated with higher educational level (OR = 0.55; 95% CI 0.39–0.75), being physically active (OR = 0.66; 95% CI 0.56–0.77), better access to medical institutions (OR = 0.67; 95% CI 0.45–0.99) and beds (OR = 0.55; 95% CI 0.37–0.80). Random Forests prioritized physical activity, disposable income, sleep duration, social security benefits, and Body Mass Index (BMI) as top county-level associated factors.

**Conclusions:**

These insights advocate optimizing medical resources, bolstering primary care, and fostering healthy lifestyles to reduce the burden of multimorbidity among older Chinese adults.

**Supplementary Information:**

The online version contains supplementary material available at https://doi.org/10.1186/s12877-026-07893-x.

## Introduction

Multimorbidity is the co-occurrence of two or more chronic conditions in an individual [1]. A recent systematic review and meta-analysis reported a global multimorbidity prevalence of 37.2%, with over half of adults aged 60 and above affected [2]. China is experiencing a rapid increase in its older adult population, with the number of people aged 60 and above reached 264.02 million by the end of 2020 [3]. This reflects a 5.4% increase compared to the sixth national census in 2010 [4], which parallels an increased incidence of chronic diseases and multimorbidity [5]. Multimorbidity poses significant individual and societal burdens, including functional decline [6, 7], diminished quality of life [8, 9], increased healthcare expenses in older adults [10–12], alongside catastrophic health expenditures for families [13, 14], and even premature mortality [15–17].

Research on multimorbidity has expanded substantially in both developed and developing nations. Although research on multimorbidity has advanced globally, studies remain relatively scarce in China, a country experiencing rapid population aging, particularly about regional disparities. First, previous studies on medical resources have primarily focused on individual healthcare utilization and expenditures [12, 18]. For instance, participants are commonly asked, ‘In the past year, how often have you visited healthcare facilities?’ Few studies have examined how medical resources in participants’ living areas, such as the availability of healthcare facilities, affect multimorbidity. Second, the application of machine learning in public health and medicine is becoming increasingly prevalent [19–25]. For example, Random Forests (RF) have demonstrated robust performance in modeling multimorbidity [23]. However, little research in China has employed machine learning to explore the county-level associated factors of multimorbidity among older adults. Third, due to significant heterogeneity in samples, definitions, qualifying diseases, and analytical methods, previous studies on multimorbidity prevalence and patterns have exhibited substantial variability. In China, reported multimorbidity prevalence ranges from 5.13% to 69.5% [12, 26–28], underscoring the need for more comprehensive data to better elucidate multimorbidity trends and characteristics.

Therefore, by utilizing comprehensive data from the 2020 China Longitudinal Aging Social Survey (CLASS) and regional Statistical Communiqués, this study aimed to investigate the prevalence and patterns of multimorbidity among older adults in China. Furthermore, a generalized linear mixed model (GLMM) and a RF model were employed to identify individual-level correlates and assess the relative importance of countryside/community level associated factors, respectively.

## Methods

### Data sources and collection

This cross-sectional study draws upon data from the 2020 wave of the CLASS, an ongoing, large-scale research initiative. The baseline survey, conducted between August and October 2014, spanned 28 provinces (excluding Hong Kong, Taiwan, Macau, Hainan, Xinjiang, and Tibet). Follow-up surveys are conducted biennially and focus on individuals aged 60 and above, with the aim of understanding the evolving patterns of aging in China. The CLASS employed a stratified, multistage probability sampling method, utilizing the China Social Survey Network (CSSN) to facilitate in-person data collection by trained personnel. To account for this complex survey design and the clustering of participants within regions, we explicitly incorporated village/community-level random effects into our statistical models. The mixed-effects modeling approach effectively adjusts for the hierarchical data structure, thereby mitigating potential biases in standard errors and effect estimates. Comprehensive details on the survey design and execution are available in previous publications [29, 30].

Data regarding the number of medical and health institutions, beds in these facilities, and per capita disposable income were sourced from the 2020 Statistical Communiqué on National Economic and Social Development for each city. City-level population data were extracted from the seventh national population census bulletin.

### Dependent variable

The dependent variable in this study was multimorbidity, defined as the co-occurrence of two or more chronic conditions within an individual [1]. For the GLMM, multimorbidity was treated as a binary outcome (presence vs. absence). Conversely, in the RF model, the regional prevalence of multimorbidity at the countryside/community level was analyzed as a continuous variable. Multimorbidity was evaluated based on a predefined list of 22 chronic conditions derived from the standard CLASS questionnaire. These specific conditions were selected because they capture a comprehensive spectrum of the most prevalent and burdensome physiological, sensory, and cognitive diseases affecting the aging Chinese population. The 22 conditions included: hypertension, arthritis or rheumatism, heart disease, cervical or lumbar spondylosis, diabetes or elevated blood sugar, osteoporosis, gastroenteritis or other digestive system diseases, chronic respiratory disease, cerebrovascular disease, deafness, glaucoma/cataract, prostatic diseases, nervous system disease, urinary system diseases, liver disease, kidney disease, breast diseases, Parkinson’s disease, dementia, tuberculosis, reproductive system diseases, and cancer/malignancy. Chronic disease data were based on self-reports obtained during in-person interviews, where participants were asked, “What chronic diseases do you suffer from?” To maximize data reliability, the CLASS project utilized a rigorous three-tier quality control system. All data were collected by professionally trained interviewers using a standardized questionnaire developed by Renmin University of China, under strict on-site supervision facilitated by the CSSN.

### Independent variables

The independent variables were categorized into three major groups: sociodemographic characteristics, lifestyle characteristics and medical resources.

### Sociodemographic characteristics

The sociodemographic characteristics included age, gender, educational level, marital status, social security benefits received and per capita disposable income. Age was treated as a continuous variable or discretized into three categories: 60–68, 69–78, 79–98. Educational level was divided into four groups: illiterate, primary school and below, secondary school, and junior college and above. Marital status was classified as either married or unmarried (widowed, divorced, or single). The social security benefits received included eight types: basic pension insurance for enterprise employees; pension insurance benefits for government agencies and public institutions; social endowment insurance for urban and rural residents; minimum living allowance or poverty relief fund; old age allowance; subsidies for home-based older adults care services; rural family planning rewards and assistance funds and other government assistance. This variable was divided into four groups based on the number of social security benefits received: None, 1, 2, ≥ 3. Per capita disposable income was categorized according to whether it exceeded the national average disposable income for China in 2020 (¥32,189).

### Lifestyle characteristics

The lifestyle characteristics included sleep duration, smoking status, Body Mass Index (BMI), and physical activity level. Sleep duration was categorized as sufficient or insufficient, based on participants’ self-reported average sleep per night over the past three months. Sleeping at least 7 h per night was considered sufficient, while less than 7 h was deemed insufficient [31]. Smoking status was classified as smoker or non-smoker, where smokers were defined as individuals who had smoked more than 100 cigarettes in their lifetime. BMI was calculated using participants’ height and weight scales. Participants were categorized into thin (BMI < 18.5 kg/m^2^), normal weight (18.5 ≤ BMI ≤ 23.9 kg/m^2^), overweight (23.9 < BMI ≤ 27.9 kg/m^2^), and obese (BMI > 27.9 kg/m^2^). Physical activity levels were divided into two groups: active or inactive. The time each participant spent on physical activity per week was quantified using Metabolic Equivalent of Task (MET) minutes, calculated by multiplying the duration of each activity type by its corresponding MET value. Inactive status was assigned to individuals failing to meet the criteria based on the International Physical Activity Questionnaire group. Specifically, individuals were deemed inactive if their weekly physical activity did not meet at least one of the following conditions:

- Engaging in vigorous-intensity physical activity for a minimum of 20 minutes on 3 or more days per week.

OR

b) Participating in moderate-intensity physical activity and/or low-intensity physical activity for a minimum of 30 minutes on 5 or more days per week.

OR

c) Involvement in any combination of low-intensity physical activity, moderate-intensity physical activity, or vigorous-intensity physical activity on 5 or more days, resulting in a cumulative MET-minutes/week at least 600.

Participants meeting any of these criteria were categorized as active.

### Medical resources

Medical resources were represented by the number of medical and health institutions per 1,000 residents and the number of beds in medical and health institutions per 1,000 residents. Both variables were categorized into quartiles: Q1 (lowest) to Q4 (highest), representing areas with the fewest to the most medical resources, respectively.

### Statistical analysis

Data analysis was conducted using descriptive statistics, Chi-squared tests, the least absolute shrinkage and selection operator (LASSO) regression, GLMM, and RF. Missing data were handled using a complete case analysis. Continuous variables (e.g., age) were summarized as means with Standard Deviations (SD), while categorical variables were presented as frequencies and percentages. Group differences among categorical variables were evaluated using Chi-squared tests. To optimize feature selection and mitigate potential multicollinearity, LASSO regression was applied prior to the main modeling. Subsequently, GLMM incorporating village/community-level random effects were constructed to identify individual-level correlates of multimorbidity while accounting for data clustering. ArcMap software (version 10.8) was employed to visualize the geographic distribution of medical resources.

The LASSO was applied to perform variable selection and mitigate potential multicollinearity among the independent variables. Specifically, a 10-fold cross-validation was conducted, and the penalty parameter (λ) located at one standard error from the minimum cross-validation error (λ 1se) was utilized to retain variables with non-zero coefficients. During this process, categorical variables were transformed into dummy variables. Variables retained by the LASSO procedure were subsequently included in the GLMM. To account for the hierarchical data structure, the village/community identifier was included as a random effect in the model. ORs with 95% Cis were calculated for each factor in the GLMM.

The growing adoption of machine learning in public health and medicine stems from its predictive advantages [32–35]. In this study, a RF algorithm was employed to complement the individual-level GLMM by evaluating macroscopic, county-level factors associated with multimorbidity prevalence. As an ensemble learning method, RF improves prediction accuracy by aggregating multiple decision trees, reducing the risk of overfitting associated with single trees [36]. It efficiently handles complex nonlinear relationships and demonstrates strong robustness against noise and missing data. For this study, the focus was on the countryside/community level, as county-level analysis is frequently utilized in ecological health research [37]. We calculated the average values of both dependent and independent variables at the countryside/community level, which were then input into the RF model for regression analysis. Model hyperparameters were optimized using a five-fold cross-validation grid search approach via the Scikit-learn library. Based on this optimization, the final model was configured with: 100 decision trees (n_estimators = 100), a maximum depth of 30 (max_depth = 30), a minimum split sample of 2 (min_samples_split = 2), a minimum leaf sample of 1 (min_samples_leaf = 1), and bootstrap sampling enabled (Supplementary Table S1). The Root Mean Squared Error (RMSE) and R-squared (R²) were used to assess the RF model’s performance. To interpret the model and rank associated factors, we utilized permutation importance with 30 repeats. Furthermore, Shapley Additive exPlanations (SHAP) and SHAP interaction values were calculated to capture potential synergistic effects, with detailed visualization plots provided in the Supplementary Material. We implemented these modelling exercises using Python 3.8.10.

To ensure the robustness of our findings and address potential biases, four prespecified sensitivity analyses were conducted using the GLMM framework. First, to test the stability of correlates across different definitions, we redefined the multimorbidity threshold as the presence of three or more chronic conditions. Second, to address concerns regarding disease heterogeneity and clinical diagnostic consistency, we restricted the disease count to 18 core systemic conditions, excluding potentially non-systemic or controversial conditions such as tuberculosis, reproductive system diseases, gastroenteritis, and deafness. Finally, to explore the potential for reverse causality—specifically regarding the substantial association between social security benefits and multimorbidity—we performed an age-stratified analysis (60–68 years vs. ≥69 years). This stratification allowed us to evaluate whether the observed associations remained consistent across relatively younger, healthier cohorts compared to older cohorts characterized by underlying vulnerability or prevalent disability-driven benefits. Fourth, to mitigate recall bias related to cognitive impairment, a key concern for self-reported comorbidity accuracy, we restricted the analysis to participants without cognitive impairment-related conditions, excluding diseases that may compromise the accuracy of self-reported comorbidities (e.g., dementia, neurological disorders, Parkinson’s disease). This approach aimed to reduce the impact of inaccurate self-reporting in individuals with cognitive decline particularly in the oldest-old group and verify the stability of our primary associations in a cognitively unimpaired subgroup.

A *p*-value $\:<\:0.05$ was considered statistically significant throughout this study unless otherwise specified.

## Results

### Sociodemographic and lifestyle characteristics of participants

This study utilized data from the CLASS 2020 survey, which included 11,372 older adults aged 60 and above. We presented the prevalence of multimorbidity among older adults, stratified by sociodemographic factors, lifestyle characteristics, and medical resources (Table 1). The mean age of the participants was 71.6 years (SD = 6.6, range: 60–98 years). The overall prevalence of multimorbidity was 46.03%. Significant differences were observed between the non-multimorbidity and multimorbidity groups in age, gender, education level, marital status, smoking status, BMI, physical activity levels, social security benefits received, sleep duration, per capita disposable income (2020), number of medical and health institutions per 1,000 residents, and number of hospital beds per 1,000 residents (chi-square test, all *P* < 0.001).

**Table 1:** Sociodemographic, lifestyle characteristics and medical resources of participants

Characteristics	Total, *n* (%)	Non-multimorbidity	Multimorbidity	*P*-value	χ^2^
Total	11,372(100%)	6138(53.97%)	5234(46.03%)		
Age (years)					
Mean (SD)	71.6 (6.6)	70.6 (6.2)	72.8 (6.8)		
59–68	4,376 (38.48%)	2,750 (62.84%)	1,626 (37.16%)	< 0.001	308.02
69–78	5,099 (44.84%)	2,638 (51.74%)	2,461 (48.26%)		
79–98	1,897 (116.68%)	750 (39.54%)	1,147 (60.46%)		
Sex					
Male	5,737 (50.45%)	3,303 (57.57%)	2,434 (42.43%)	< 0.001	60.37
Female	5,635 (49.55%)	2,835 (50.31%)	2,800 (49.69%)		
Educational level					
Illiterate	2,668 (23.46%)	1,358 (50.90%)	1,310 (49.10%)	< 0.001	24.67
Primary school and below	4,673 (41.09%)	2,489 (53.26%)	2,184 (46.74%)		
Secondary school	3,764 (33.10%)	2,135 (56.72%)	1,629 (43.28%)		
Junior college and above	267 (2.35%)	156 (58.43%)	111 (41.57%)		
Marital status					
Married	8,568 (75.34%)	4,786 (55.86%)	3,782 (44.14%)	< 0.001	49.67
Unmarried	2,804 (24.66%)	1,352 (48.22%)	1,452 (51.78%)		
Smoking					
Yes	3,168 (27.86%)	1,794 (56.63%)	1,374 (43.37%)	< 0.001	12.45
No	8,204 (72.14%)	4,344 (52.95%)	3,860 (47.05%)		
BMI					
Thin	596 (5.24%)	336 (56.38%)	260 (43.62%)	< 0.001	44.93
Normal weight	7,207 (63.37%)	3,985 (55.29%)	3,222 (44.71%)		
Overweight	3,153 (27.73%)	1,653 (52.43%)	1,500 (47.57%)		
Obese	416 (3.66%)	164 (39.42%)	252 (60.58%)		
Physical activity					
Active	8,091 (71.15%)	4,554 (56.28%)	3,537 (43.72%)	< 0.001	60.24
Inactive	3,281 (28.85%)	1,584 (48.28%)	1,697 (51.72%)		
Number of social security benefits enjoyed					
None	744(6.54%)	561(75.40%)	183(24.60%)	< 0.001	327.70
1	4,659 (40.97%)	2,781 (59.69%)	1,878 (40.31%)		
2	5,664 (49.81%)	2,675 (47.23%)	2,989 (52.77%)		
≥ 3	305 (2.68%)	121 (39.67%)	184 (60.33%)		
Sleep duration					
Sufficient	8,906 (78.32%)	4,882 (54.82%)	4,024 (45.18%)	< 0.001	11.73
Insufficient	2,466 (21.68%)	1,256 (50.93%)	1,210 (49.07%)		
Per capita disposable income in 2020					
Did not exceed	5,791 (50.92%)	3,256 (56.23%)	2,535 (43.77%)	< 0.001	24.06
Exceeded	5,581 (49.08%)	2,882 (51.64%)	2,699 (48.36%)		
Number of medical and health institutions per 1000 residents					
Q1(The least)	3,840 (33.77%)	1,879 (48.93%)	1,961 (51.07%)	<0.001	120.94
Q2	2,538 (22.32%)	1,284 (50.59%)	1,254 (49.41%)		
Q3	2,413 (21.22%)	1,391 (57.65%)	1,022 (42.35%)		
Q4(The most)	2,581 (22.70%)	1,584 (61.37%)	997 (38.63%)		
Number of beds in medical and health institutions per 1000 residents					
Q1(The least)	3,151 (27.71%)	1,431 (45.41%)	1,720 (54.59%)	<0.001	148.91
Q2	2,748 (24.16%)	1,665 (60.59%)	1,083 (39.41%)		
Q3	2,789 (24.53%)	1,525 (54.68%)	1,264 (45.32%)		
Q4(The most)	2684(23.60%)	1517(56.52%)	1167(43.48%)		

### Prevalence of multimorbidity

**Fig. 1:**
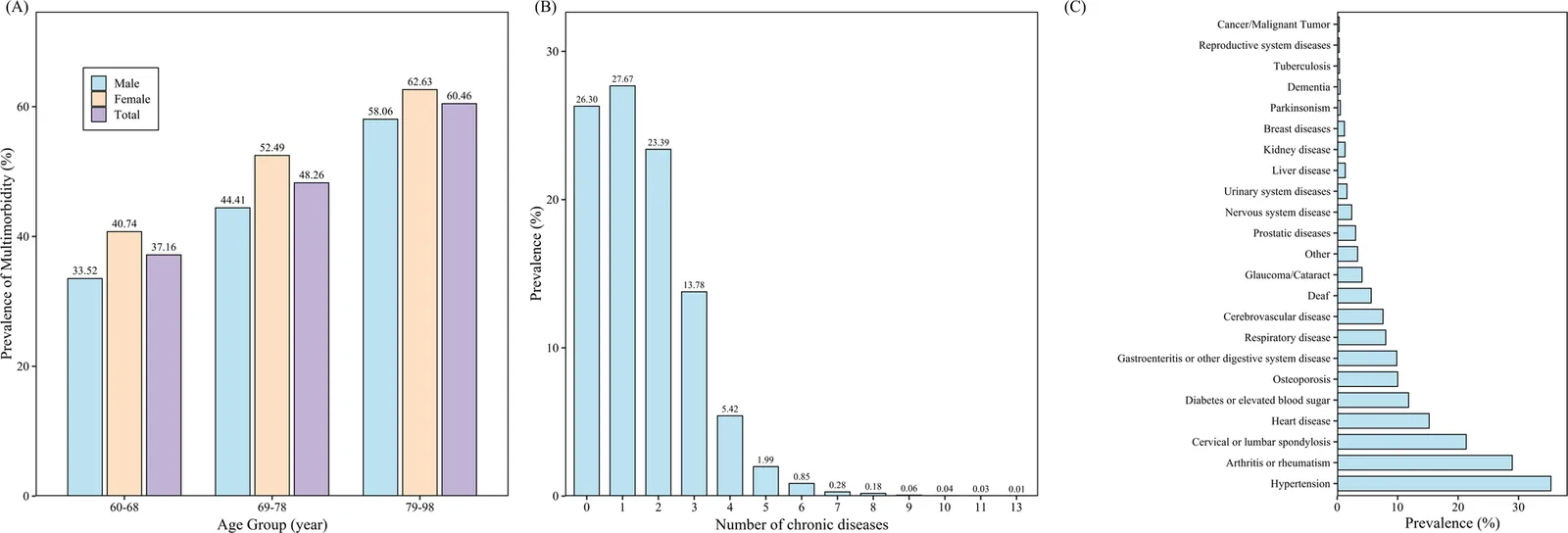
The prevalence of multimorbidity by age and gender (**A**), the prevalence of the number of chronic conditions (**B**), and the prevalence of the 23 chronic condition categories in the CLASS (**C**)

We analyzed the prevalence of multimorbidity by age, gender, number of chronic conditions, and types of chronic conditions (Fig. 1). The prevalence of multimorbidity quantitatively increased with advancing age across both genders, rising from 37.16% in the 60–68 age group to 48.26% in the 69–78 age group, and reaching 60.46% in the 79–98 age group (*P* < 0.001). Within all corresponding age groups, the prevalence was consistently higher in females compared to males; specifically, 40.74% versus 33.52% in the 60–68 group, 52.49% versus 44.41% in the 69–78 group, and 62.63% versus 58.06% in the 79–98 group (Fig. 1A). The number of chronic diseases reported per participant ranged from 0 to 13. According to the questionnaire, participants were evaluated for 22 specific chronic diseases along with an additional “Other” category (representing self-reported unlisted conditions, resulting in 23 categories analyzed). Approximately 26.30% of participants reported no chronic diseases, while 46.03% reported having multimorbidity (two or more chronic diseases, Fig. 1B). The five most common chronic diseases were hypertension (35.36%), arthritis or rheumatism (28.96%), cervical or lumbar spondylosis (21.34%), heart disease (15.21%), and diabetes or elevated blood sugar (11.82%, Fig. 1C).

### Distribution of medical resources

**Fig. 2:**
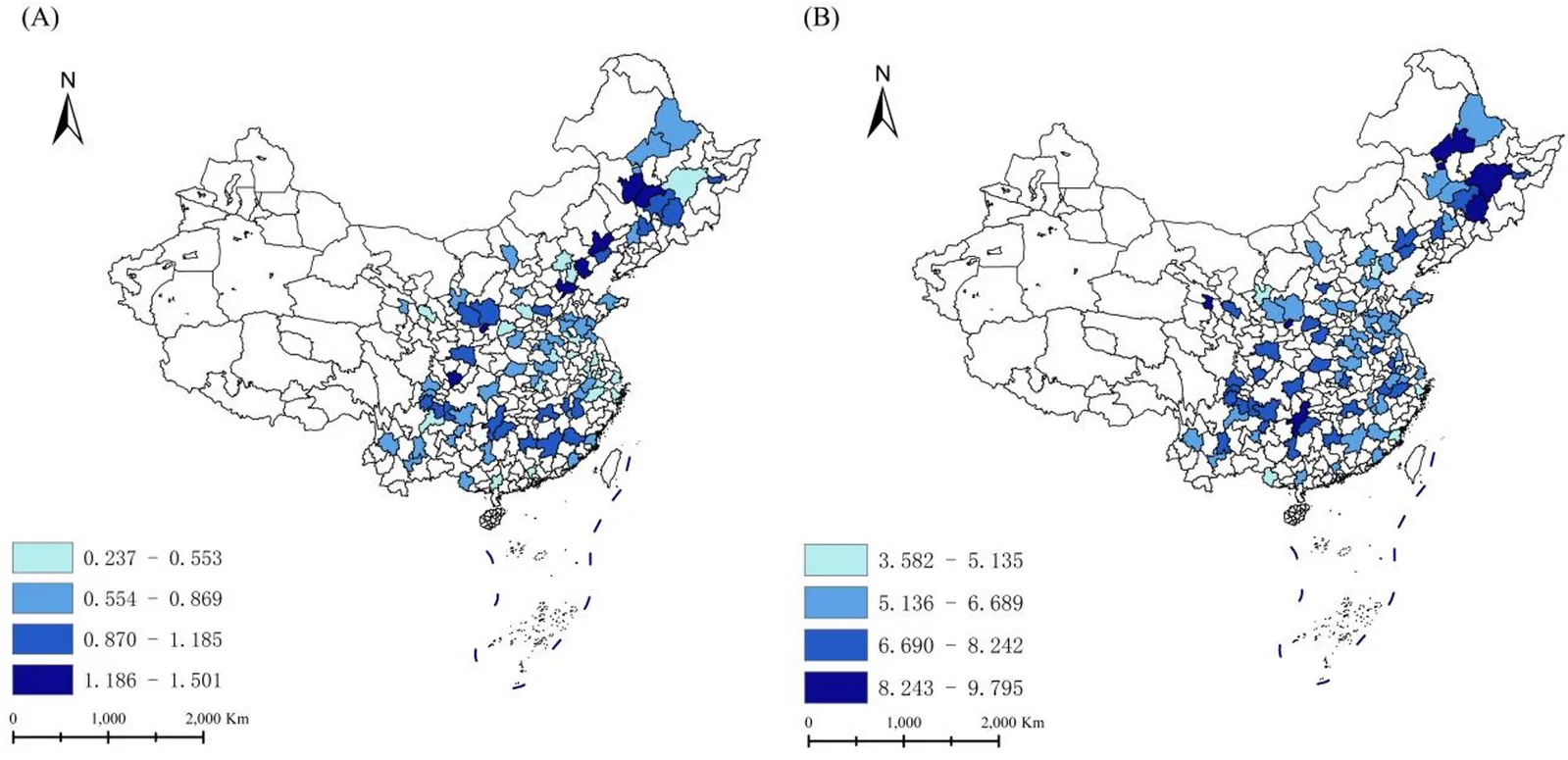
Geospatial distribution of the medical resources per 1,000 residents in China in 2020, **A** The number of medical and health institutions per 1,000 residents in China (Except Chongqing), **B** The number of beds in medical and health institutions per 1,000 residents in China (Except Chongqing)

Geospatial distribution maps of medical resources per 1,000 residents were constructed based on the 2020 Statistical Communiqués on National Economic and Social Development for each surveyed city (Fig. 2). Because Chongqing is a direct-administered municipality with a vast administrative area of approximately 82,400 km² and a population exceeding 32 million, a scale equivalent to an entire province or a medium-sized country, it exhibits significant internal regional variations; thus, it was divided into more detailed subdivisions (Supplementary Figure S1). The results revealed substantial regional disparities in healthcare infrastructure density. The number of medical and health institutions per 1,000 residents ranged from 0.237 in coastal megacities with extremely high population density (e.g., Shanghai) to 1.501 in inland resource-limited cities (e.g., Chaoyang). Similarly, hospital bed availability per 1,000 residents varied from 3.582 in major coastal metropolises (e.g., Shenzhen) to 9.795 in inland urban centers (e.g., Tongchuan). Notably, less developed inland areas exhibited higher per capita resource density compared to economically developed first-tier coastal cities. This paradoxical phenomenon is primarily attributed to the massive population concentration in megacities, which effectively dilutes the per capita availability of medical resources despite a higher absolute volume of facilities. Overall, substantial disparities in the distribution of medical resources across regions in China are evident. This diversity highlights the uneven development of healthcare systems across regions and the unique healthcare needs of different population groups.

### Factors associated with multimorbidity at the individual level

**Fig. 3:**
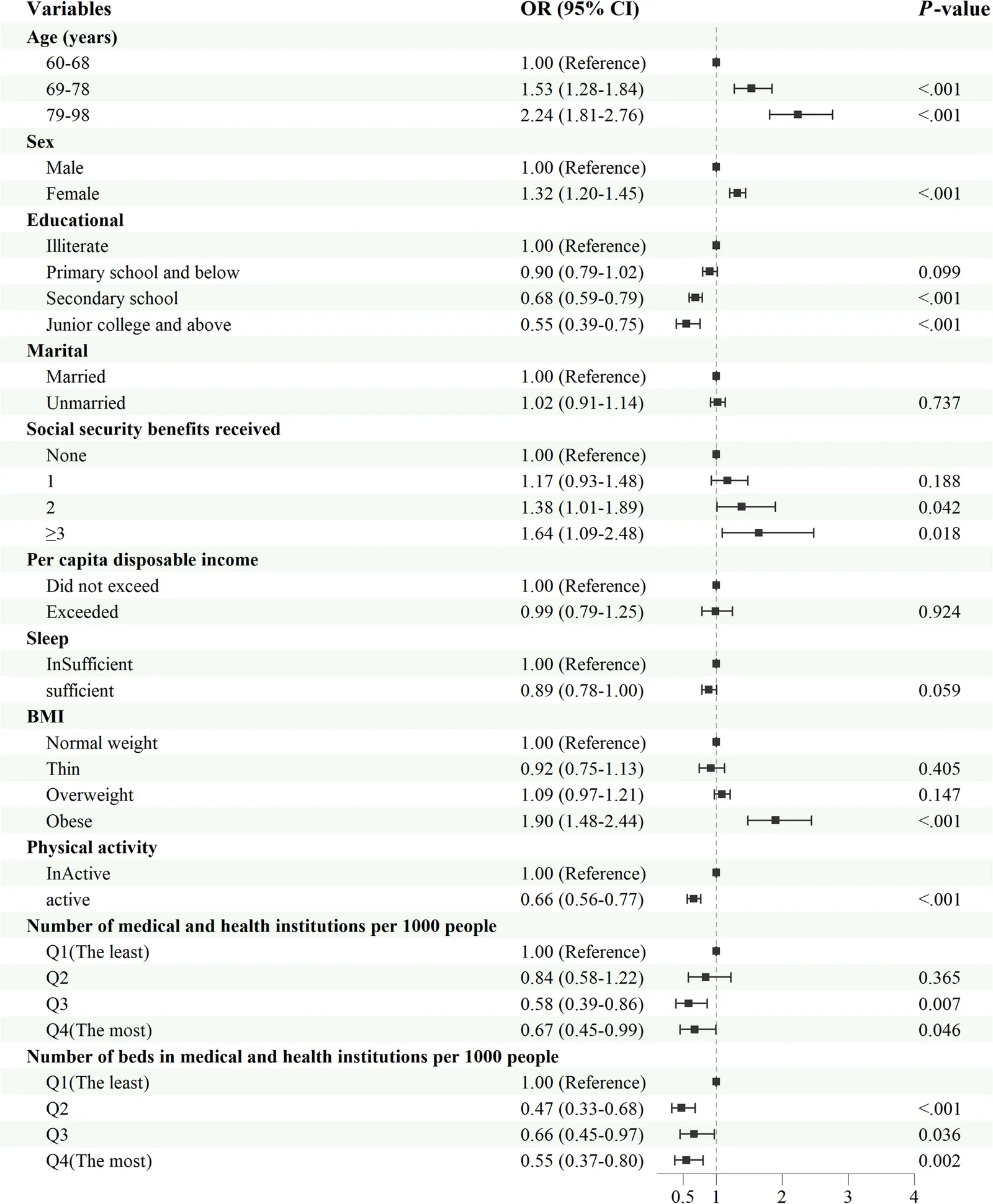
Generalized linear mixed model analysis of the factors associated with multimorbidity

Based on the LASSO regression, 11 of the 12 candidate variables were retained in the final model (Supplementary Figure S2), while smoking was excluded. This 10-fold cross-validated selection process inherently protects the final model against overfitting and multiple testing biases, ensuring the robustness and generalizability of the subsequent findings. We then performed GLMM to identify the factors associated with multimorbidity at the individual level, using the presence or absence of multimorbidity as the dependent variable. The independent variables included sociodemographic and lifestyle characteristics, as well as regional medical resources per 1,000 residents (Fig. 3). The GLMM was subsequently performed to identify individual-level correlates of multimorbidity. Model discrimination was evaluated using the conditional receiver operating characteristic (ROC) curve, which incorporates both the fixed effects and the village/community-level random effects. This approach yielded a robust Area Under the Curve (AUC) of 0.83 (Supplementary Figure S3). In terms of age, the odds of multimorbidity among adults aged 69–78 were 1.53 times that of the reference population aged 60–68 (OR = 1.53, 95% CI: 1.28–1.84), while the odds in those aged 79–98 were 2.23 times that of the 60–68 age group (OR = 2.23, 95% CI: 1.81–2.76). Females had higher odds of multimorbidity than males (OR = 1.31, 95% CI: 1.18–1.46). Participants receiving three or more social security benefits had higher odds of multimorbidity compared to those with no social security benefits (OR = 1.64, 95%CI: 1.09–2.48). Regarding lifestyle characteristics, physically active participants had lower odds of multimorbidity (OR = 0.66, 95% CI: 0.56–0.77). Obesity was associated with higher odds of multimorbidity (OR = 1.90, 95% CI: 1.48–2.44). Concerning medical resources, participants residing in areas with higher numbers of medical and health institutions per 1,000 people had lower odds of multimorbidity compared to those in the area with the least number (Q4 vs. Q1: OR = 0.67, 95% CI: 0.45–0.99). Similarly, participants in areas with higher numbers of medical beds per 1,000 people had lower odds of multimorbidity compared to those in the area with the least number (Q4 vs. Q1: OR = 0.55, 95% CI: 0.37–0.80). In the final GLMM model, marital status was not significantly associated with multimorbidity. In summary, factors associated with lower odds of multimorbidity included higher physical activity levels, greater access to medical and health institutions, and an increased number of beds in medical and health institutions.

### Random forest models identified key predictive factors for regional prevalence of multimorbidity

**Fig. 4:**
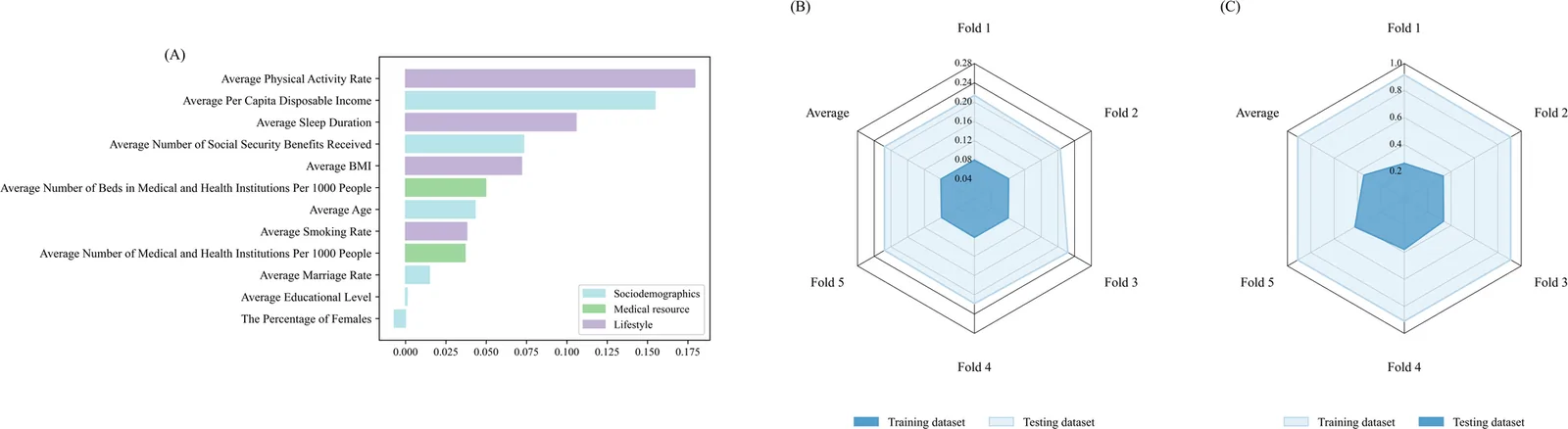
Prediction result and evaluation index of RF model, **A** RF ranking results for factors influencing the prevalence of multimorbidity, **B** and **C** Distribution of RMSE (**B**) and R² (**C**) values across the 5-fold cross-validation iterations

We employed RF model to investigate the relative importance of factors influencing the regional prevalence of multimorbidity (Fig. 4). The results indicated that in units at the countryside/community level, average physical activity rate, average per capita disposable income, average sleep duration, average number of social security benefits received, and average BMI were the top five predictors of regional multimorbidity prevalence (Fig. 4A). This finding further highlighted that the significance of regional medical resources and lifestyle factors surpasses that of sociodemographic characteristics, such as the percentage of females and average marriage rate, in predicting the regional prevalence of multimorbidity. To rigorously evaluate the regional RF model’s performance and generalizability, we reported the RMSE (Fig. 4B) and R² metrics using 5-fold cross-validation, which provides a robust assessment of model stability across independent data subsets. The model demonstrated excellent stability across all 5 validation folds, with minimal variation in performance metrics between iterations, indicating consistent predictive behavior across different regional subgroups. Critically, the primary objective of our RF analysis was not to achieve precise point prediction of regional multimorbidity rates, but rather to identify the relative importance of modifiable regional determinants and uncover their complex non-linear relationships. Furthermore, to provide interpretable context for the model’s predictions and clarify the directionality and magnitude of each factor’s impact, we employed Shapley Additive exPlanations (SHAP) analysis (Supplementary Figure S4). The SHAP summary plot revealed that higher average per capita disposable income, greater average number of social security benefits received, and higher average BMI were positively associated with regional multimorbidity prevalence, while higher average physical activity rate was negatively associated, consistent with our individual-level GLMM findings in terms of association direction. To further explore potential structural synergies between factors, we conducted pairwise SHAP interaction analysis and presented the full interaction matrix in Supplementary Figure S5. In the main text, we highlight the two most clinically and policy-relevant interactions: the strong positive interaction between average per capita disposable income and average number of beds in medical and health institutions per 1000 people, indicating that the combined effect of regional economic development and healthcare resource supply on multimorbidity prevalence is greater than the sum of their individual effects. The second notable interaction was between average physical activity rate and average sleep duration, suggesting that the protective effect of regular physical activity against multimorbidity is further enhanced by adequate sleep duration, underscoring the importance of integrated healthy lifestyle interventions for older adults. These interactions highlight the combined influence of economic, healthcare, and lifestyle factors in shaping regional multimorbidity disparities.

### Sensitivity analyses

To rigorously validate the robustness of our individual-level findings, we performed four sensitivity analyses. First, elevating the multimorbidity diagnostic threshold to ≥ 3 chronic conditions yielded highly consistent associations across all major socio-demographic and lifestyle variables (Supplementary Figure S6). Second, after excluding deafness and reproductive system diseases from the multimorbidity definition, the direction and magnitude of the primary effect estimates remained substantively unchanged (Supplementary Figure S7). Third, an age-stratified analysis revealed that the pronounced association between receiving ≥ 3 social security benefits and multimorbidity was predominantly driven by the older demographic (≥ 69 years: OR = 1.86, 95% CI: 1.31–2.63, *P* < 0.001), whereas it was attenuated to marginal significance in the relatively younger group (60–68 years: *P* = 0.057) (Supplementary Table S2). Finally, after restricting the analysis to participants without cognitive impairment-related conditions (excluding diseases like dementia, neurological disorders, and Parkinson’s disease), the primary associations remained substantively consistent with the main analysis (Supplementary Figure S8). Key correlates such as age, sex, educational attainment, BMI, physical activity, social security benefits, and medical resource availability retained their original direction of effect and statistical significance, with no meaningful shifts in effect magnitude. This confirms that potential recall bias from cognitive impairment did not substantially alter our core findings. Collectively, these analyses underscore the robustness of our primary findings across varying diagnostic criteria, while clarifying those specific indicators—such as multiple social security benefits—reflect underlying vulnerability or an advanced disease burden.

## Discussion

With accelerating global aging, multimorbidity has emerged as a critical public health challenge. This study is the first to examine the factors associated with multimorbidity among older Chinese adults at both individual and regional levels, using nationally representative CLASS 2020 data and machine learning techniques. Among 11,372 participants aged 60 and older from 28 provinces, 46.03% reported multimorbidity, with prevalence increasing with age and higher among females. Significant regional disparities in medical resources per 1,000 residents highlight uneven healthcare allocation across China. At the individual level, active physical activity and greater access to medical institutions and beds were associated with lower odds of multimorbidity. Regionally, Random Forest models identified physical activity, per capita disposable income, sleep duration, social security benefits, and BMI as key predictors, with lifestyle and medical resources outweighing sociodemographic factors. These findings emphasize the critical role of equitable resource distribution and healthy lifestyles In mitigating multimorbidity.

Approximately 46% of older adults in our sample reported multimorbidity, consistent with previous studies [38, 39], though some have reported higher or lower estimates due to differences in age, disease definitions, and measurement approaches [12, 28]. Our estimate aligns broadly with global data showing that over half of the population aged 60 years and above experience multimorbidity (51.0%, 95% CI = 44.1–58.0%) [2]. This highlights the substantial burden of multimorbidity worldwide, and our inclusion of 22 chronic conditions provides valuable reference data for future studies.

Both age and gender were significantly associated with multimorbidity. The prevalence increased with advancing age, likely reflecting natural immune decline and accumulated exposure to factors associated with chronic diseases. Females were more likely to report multimorbidity than males, possibly due to higher health awareness and a greater likelihood of reporting symptoms [40–42]. Another reason may be that the gains in healthy and disability-free life expectancy were greater for males than females [43]. These results align with studies using CHARLS and LASI data, which also noted rising prevalence with age and higher rates among females [44, 45].

While wealthier adults generally have more diagnostic opportunities [46], receiving multiple social security benefits (OR = 1.64) requires a different interpretation. In China, benefits like the minimum living allowance and care subsidies are means-tested or disability-based. Therefore, this association does not reflect affluence; rather, it indicates reverse causality, where sick and highly vulnerable individuals are the ones qualifying for these safety nets. These findings align with Wei-Quan Lin et al. [47], who found that individuals with medical insurance were more likely to experience multimorbidity. Similar patterns have been observed in other low- and middle-income countries [42, 45], contrasting with findings from high-income settings where affluent groups tend to have lower multimorbidity [48]. This difference may reflect distinct stages of epidemiological transition.

In contrast to these complex patterns, several individual-level factors aligned with conventional epidemiological expectations. Higher educational level, for instance, was associated with lower odds of multimorbidity. Furthermore, physically active older adults had significantly lower rates of multimorbidity, while obesity was associated with higher odds of multimorbidity.

Similar expected patterns were also observed at the regional level. Regionally, physical activity, sleep duration, and BMI ranked among the top determinants of multimorbidity, underscoring the importance of healthy lifestyle behaviors [49–53]. These findings echo global evidence linking inactivity, poor sleep, and excess weight with greater chronic disease burden.

However, the impact of macroscopic healthcare resources presented a more complex picture. We also observed that greater availability of medical institutions and hospital beds was inversely associated with multimorbidity. While greater healthcare access typically increases detection rates (detection bias), our inverse association suggests a dominant prevention and management effect: improved primary care enables earlier intervention, preventing single conditions from progressing into multimorbidity. However, we cannot rule out reverse causality at the macroscopic level, where high-burden regions receive compensatory medical resources. Notably, significant disparities in the distribution of medical resources across regions in China were evident, consistent with the findings of a prior study [54]. Our results align with Mu et al. [55], who showed that access to medical support and community facilities were inversely associated with multimorbidity. Given that individuals with multimorbidity require more outpatient and inpatient services [18, 56], equitable healthcare resource distribution remains vital for this population.

To our knowledge, this is the first study to integrate nationally representative data and machine learning to examine multimorbidity determinants at both individual and regional levels in China. Integrating GLMM and RF provided complementary insights. For instance, per capita disposable income ranked highly in RF despite lacking independent linear significance in GLMM. The discrepancy occurs because GLMM estimates individual-level adjusted independent associations, while Random Forest evaluates county/community-level ecological relationships involving non-linear and interactive effects. Per capita disposable income may not show a significant linear effect at the individual level but may act as an important contextual factor at the area level.

Our findings provide actionable insights for public health policy. We recommend improving healthcare resource allocation in rural and economically disadvantaged areas, upgrading primary healthcare, and promoting health education and physical activity programs for older adults. Strengthening social security systems and adopting digital health tools such as AI-assisted chronic disease management platforms can further improve continuity of care, especially in underserved regions.

Nevertheless, this study has several limitations. First, the cross-sectional design precludes causal inference and introduces potential reverse causality, particularly regarding the associations of social security benefits and macroscopic medical resources with multimorbidity. Second, reliance on self-reported chronic conditions may introduce recall bias and differential misclassification, especially underreporting due to cognitive decline among the oldest-old. Third, ecological analyses may be subject to ecological fallacy. Fourth, following established methodological precedents in previous studies utilizing this repository, we utilized complete case analysis assuming the data were missing completely at random because the database only releases a pre-cleaned dataset. Although the lack of raw missing data precludes formal testing of this assumption, the effective sample’s demographic alignment with national census data supports its continued representativeness. Finally, survivor bias may affect estimates among the oldest age groups. To rigorously mitigate these limitations, we conducted a targeted series of sensitivity analyses. To address the reverse causality concerns, we performed an age-stratified analysis. This confirmed that the association between receiving multiple social security benefits and multimorbidity was predominantly driven by the older demographic, reflecting underlying vulnerability and an advanced disease burden rather than affluence. To minimize the impact of self-reporting and recall biases, we restricted the analysis to participants without cognitive impairment-related conditions, confirming that cognitive decline did not substantially alter our core findings. While these methodological approaches successfully address reporting biases and clarify complex cross-sectional associations, they cannot fully eliminate the inherent limitations of ecological fallacy, missing data handling, or survivor bias. Therefore, future longitudinal cohort studies are required to establish true causal pathways and further validate the macroscopic determinants of multimorbidity.

## Conclusion

This study provides the first comprehensive analysis of multimorbidity prevalence and its determinants among older Chinese adults, using nationally representative CLASS 2020 data. We found a substantial prevalence of multimorbidity among older Chinese adults, driven primarily by advancing age, female gender, and socio-economic vulnerability. Our analyses reveal that healthy lifestyles and equitable healthcare allocation are strongly associated with lower multimorbidity risks, outweighing traditional demographic factors. These findings provide a solid foundation for public health strategies to address China’s escalating multimorbidity burden through equitable primary care distribution and community-based lifestyle interventions.

## Supplementary Information

Supplementary Material 1.

## Data Availability

The analyzed results during the current study are available from the corresponding author on reasonable request.

## Abbreviations

GLMM: Generalized Linear Mixed Model
RF: Random Forests
SD: Standard Deviations
OR: Odds Ratios
CI: Confidence Interval
BMI: Body Mass Index
CLASS: China Longitudinal Aging Social Survey
CSSN: China Social Survey Network
MET: Metabolic Equivalent of Task
LASSO: Least absolute shrinkage and selection operator
RMSE: Root Mean Squared Error
R²: R-squared
ROC: Receiver Operating Characteristic
AUC: Area Under the ROC Curve
CHARLS: China Health and Retirement Longitudinal Study
LASI: Longitudinal Aging Study in India
