# Supplementary material for: Prevalence and determinants of multimorbidity in older Chinese adults: a nationwide cross-sectional study using CLASS data
Source: BMC Geriatr. 2026 Jul 6;26:1228. doi: 10.1186/s12877-026-07893-x (PMC13625285; doi:10.1186/s12877-026-07893-x)
Supplement: Supplementary file 1 [file 12877_2026_7893_MOESM1_ESM.pdf]

Prevalence and Determinants of Multimorbidity in Older Chinese Adults: A Nationwide Cross-Sectional Study Using CLASS Data

Yi Li[1,†], Chenxi Zhao[2,3,†], Xinhao Zhuang[1,†], Songbo Jia[1], Xuelin Zhang[1], Liming Yan[4], Huaguang Zheng[5], Yiqun Pang[6], Bing Zhan[1], Xiaotian Li[1], Jinxi Zhang[1], John S. Ji[7], Yanjun Guo[8,*], Bingxiang Xu[3,*], Zhixiong Zhou[1,*] and Qingzhu Yang[1,*]

[1] Capital University of Physical Education and Sports, Beijing, 100191, China

[2] School of Sciences, Hebei University of Technology, Tianjin, 300401, China

[3] Key Laboratory of Hebei Province for Molecular Biophysics, Institute of Biophysics, School of Health Science & Biomedical Engineering, Hebei University of Technology, Tianjin, 300130, China

[4] School of Medicine, Tsinghua University, Beijing, 100084, China

[5] Beijing Tiantan Hospital, Capital Medical University, Beijing, China

[6] School of Physical Education, Southwest Petroleum University, Chengdu, Sichuan Province, 610000, China

[7] Vanke School of Public Health, Tsinghua University, Beijing, 100084, China

[8] School of Public Health, Tongji Medical College, Huazhong University of Science and Technology, Wuhan, Hubei Province, 430030, China

[*]Correspondence: Qingzhu Yang (yangqingzhu@cupes.edu.cn), Zhixiong Zhou (zhouzhixiong@cupes.edu.cn), Bingxiang Xu (xubingxiang@sus.edu.cn), Yanjun Guo (yanjunguo@hust.edu.cn)

[†] These authors contributed equally: Yi Li, Chenxi Zhao, Xinhao Zhuang

Supplementary Methods

1. Variable Selection using LASSO Regression

To optimize feature selection, prevent overfitting, and mitigate potential multicollinearity among the independent variables, we applied the least absolute shrinkage and selection operator (LASSO) regression prior to constructing the Generalized Linear Mixed Model (GLMM). We conducted a 10-fold cross-validation to identify the optimal penalty parameter (λ). To prioritize a more parsimonious model with strong predictive performance, the λ value located at one standard error from the minimum cross-validation error (λ 1se) was utilized. During this procedure, all categorical variables were transformed into dummy variables. Out of 12 candidate sociodemographic and lifestyle variables, 11 were retained with non-zero coefficients. Smoking status was excluded during this penalization process. The retained variables were subsequently incorporated into the GLMM analysis (Supplementary Figure S2).

2. Random Forest Model Training and Hyperparameter Tuning

To complement the individual-level GLMM, a Random Forest (RF) algorithm was employed to evaluate the relative importance of county/community-level associated factors. The optimal hyperparameters for the RF model were determined using a comprehensive grid search strategy combined with 5-fold cross-validation, implemented via the Scikit-learn library in Python. The final optimized configuration included n_estimators = 100, max_depth = 30, min_samples_split = 2, min_samples_leaf = 1, and bootstrap = True (Supplementary Table S1). Model interpretation and feature ranking were performed using permutation importance and Shapley Additive exPlanations (SHAP) analysis.

3. Detailed Procedures for Sensitivity Analyses

To ensure the rigorousness of our individual-level findings and address potential methodological biases, we designed and executed four predefined sensitivity analyses within the GLMM framework:

1) Alternative Diagnostic Threshold: We elevated the threshold for defining multimorbidity from ≥2 to ≥3 concurrent chronic conditions to test the stability of the socio-demographic and lifestyle correlates under a stricter diagnostic criterion (Supplementary Figure S6).
2) Restriction to Core Systemic Conditions: To address concerns regarding disease heterogeneity and potential clinical diagnostic inconsistency, we restricted our disease count from the original 22 conditions to 18 core systemic conditions. Potentially non-systemic, localized, or controversial conditions—specifically deafness, reproductive system diseases, gastroenteritis, and tuberculosis—were excluded (Supplementary Figure S7).
3) Age-Stratified Analysis for Reverse Causality: To explore the potential for reverse causality—particularly regarding the positive association between receiving multiple social security benefits and higher multimorbidity odds—we stratified the cohort into relatively younger (60–68 years) and older (≥69 years) subgroups. This aimed to differentiate whether the association reflects socio-economic affluence or acts as a proxy for underlying vulnerability and disability-driven welfare eligibility (Supplementary Table S2).
4) Exclusion of Cognitive Impairment-Related Conditions: Self-reported comorbidity data can be susceptible to recall bias, especially among the oldest-old. To verify the accuracy of our primary associations, we conducted a sensitivity analysis restricting the cohort to participants without cognitive impairment-related conditions (excluding those reporting dementia, Parkinson's disease, or other severe neurological disorders) (Supplementary Figure S8).

Supplemental data

Supplementary Table S1. Optimal hyperparameters for RF algorithms. The values of bootstrap, maximum depth, minimum samples per leaf, minimum samples for split, and number of estimators optimized through a 5-fold cross-validation grid search are shown.

hyperparameters	value
bootstrap	True
max_depth	30
min_samples_leaf	1
min_samples_split	2
n_estimators	100

Supplementary Table S2. Age-stratified analysis of the association between receiving multiple social security benefits and multimorbidity. The Odds Ratios (OR), 95% Confidence Intervals (CI), and P-values derived from the Generalized Linear Mixed Model (GLMM) were shown for the relatively younger (60–68 years) and older (≥69 years) subgroups.

Age Subgroup	OR (95% CI)	*P*-value
60-68 years (Relatively younger/healthier)	0.17 (0.03-1.05)	0.057
⩾69 years (Older/more vulnerable)	1.86 (1.31-2.63)	<0.001

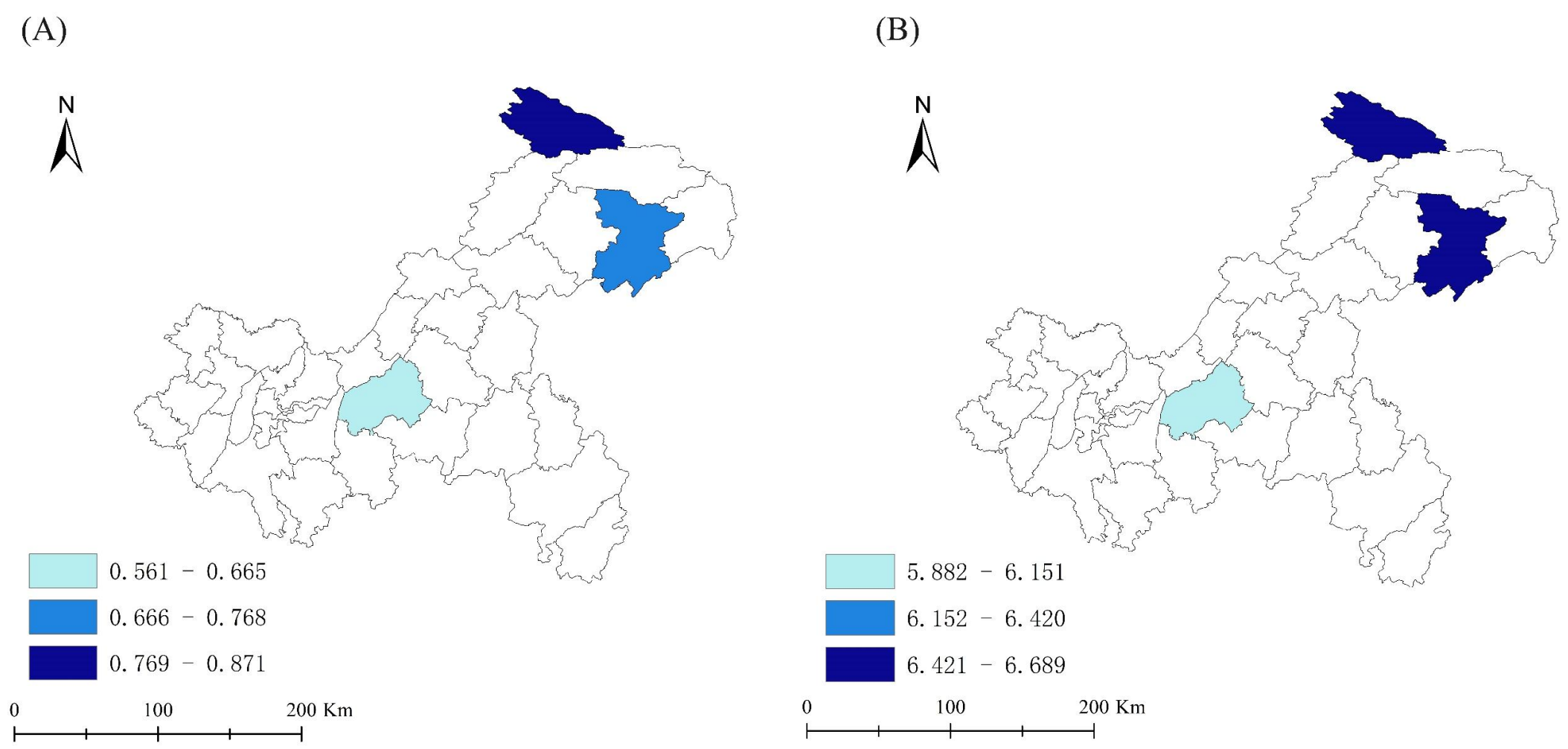

Supplementary Figure S1. Geographical distribution of the medical resources per 1,000 people in Chongqing in 2020. (A) The number of medical and health institutions per 1,000 people in Chongqing (B) The number of beds in medical and health institutions per 1,000 people in Chongqing

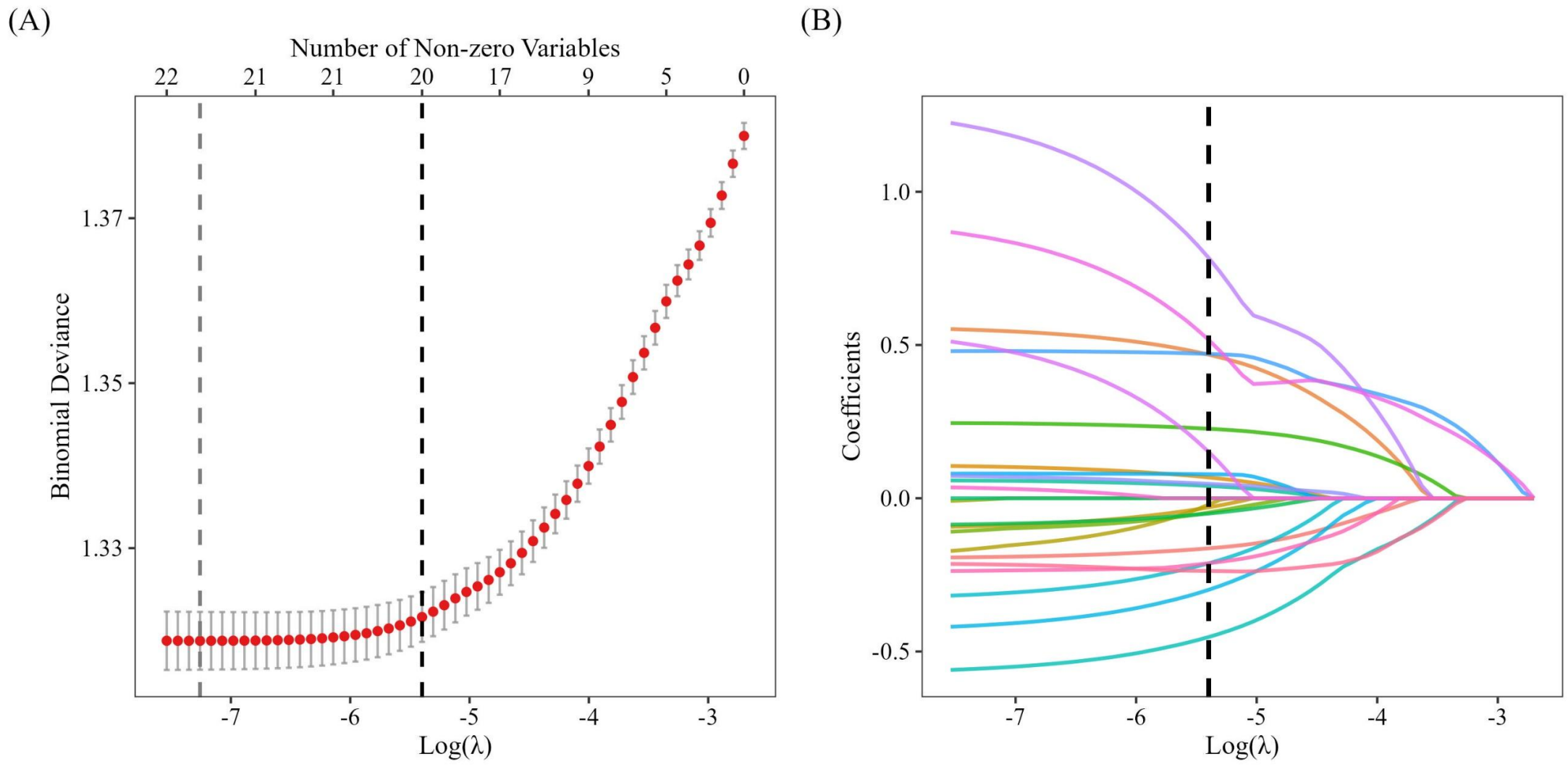

Supplementary Figure S2. LASSO regression model for variable selection. (A) The 10-fold cross-validation curve. The red dots indicate the binomial deviance values, and the grey error bars represent the standard errors across different values of the penalization parameter λ. The vertical dashed lines indicate the optimal λ values. (B) The LASSO coefficient path plot. Each colored line represents the trajectory of an individual candidate variable's coefficient as a function of log(λ).

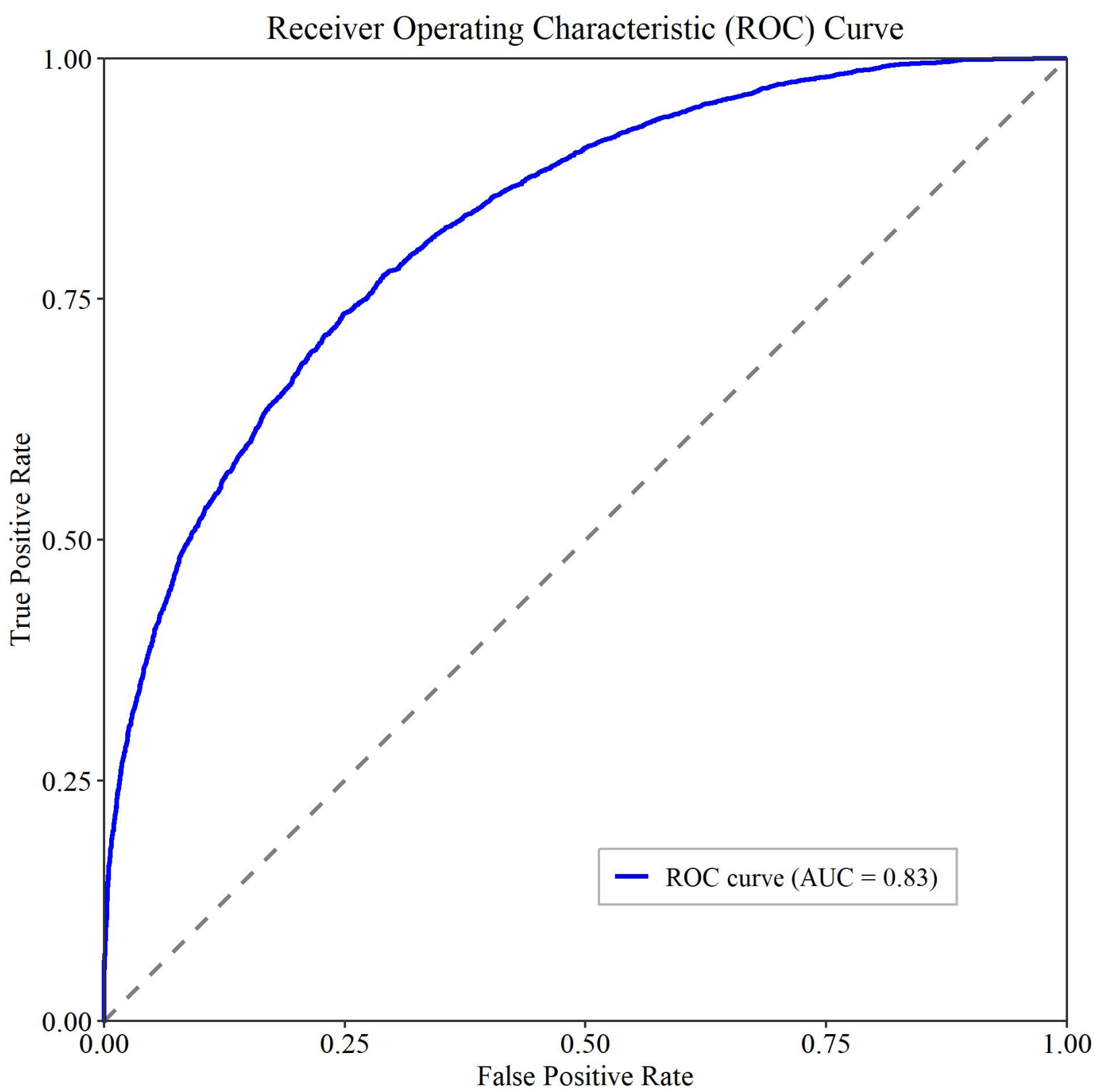

Supplementary Figure S3. ROC curves and AUC values of GLMM. The blue line represents the ROC curve for predicting individual-level multimorbidity. The dashed grey line represents the diagonal

line of no discrimination. The exact Area Under the Curve (AUC) value is marked in the legend.

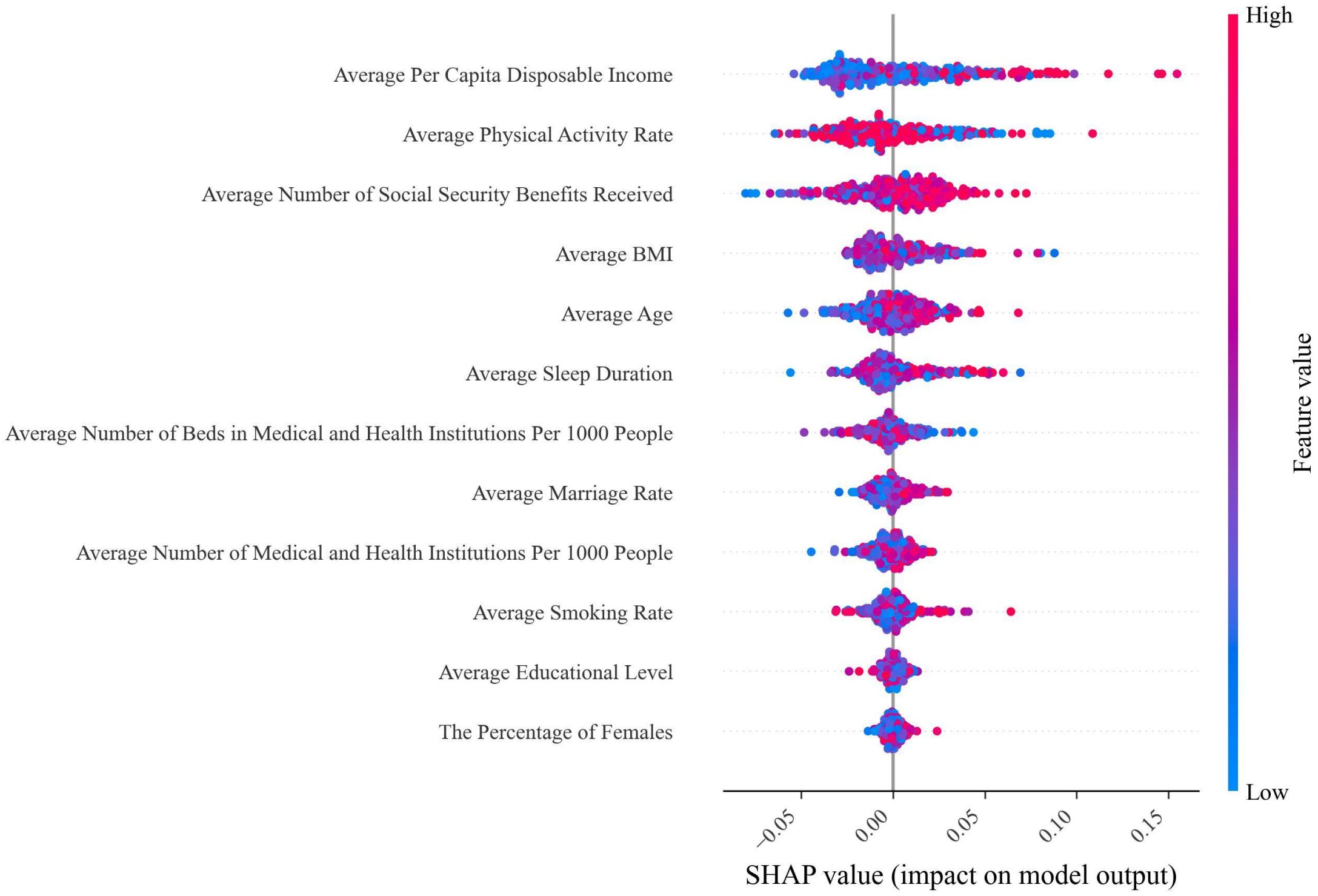

Supplementary Figure S4. SHAP value of RF. The y-axis ranks the predictive factors based on their overall importance in the Regional Random Forest model. The x-axis represents the SHAP value, indicating the impact of each feature on the model's output. Each dot represents a specific regional sample. The color gradient from blue to red represents the actual numeric value of the feature from low to high.

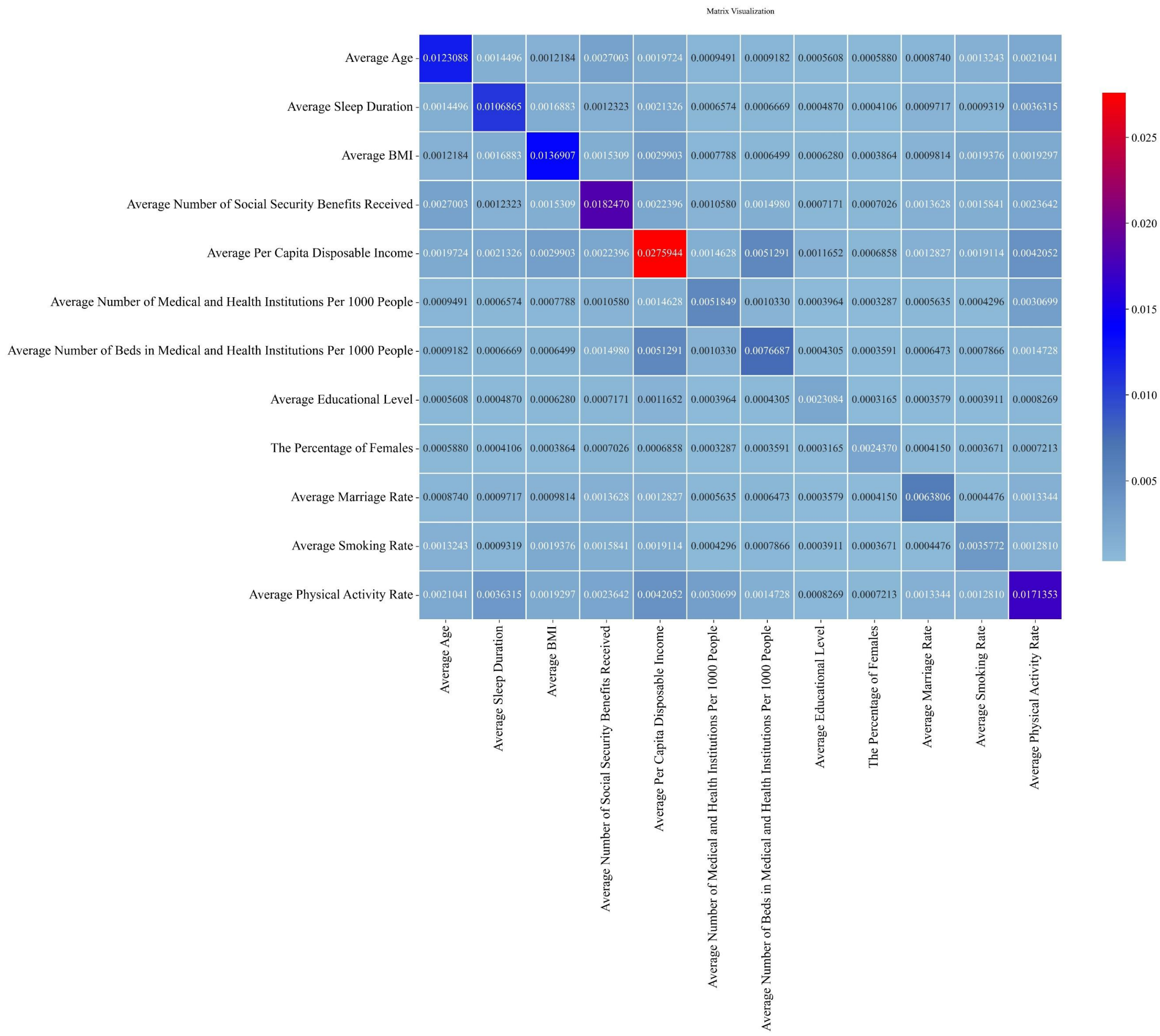

Supplementary Figure S5. Variables interaction matrix of factors influencing the prevalence of multimorbidity. The heatmap displays pairwise SHAP interaction values among factors influencing the regional prevalence of multimorbidity. The color intensity reflects the magnitude of the interaction effect. Variables are listed symmetrically on both the x-axis and y-axis.

Variables	OR (95% CI)	*P*-value
**Age (years)**		
60-68	1.00 (Reference)	
69-78	1.62 (1.29-2.04)	<.001
79-98	2.55 (1.98-3.28)	<.001
**Sex**		
Male	1.00 (Reference)	
Female	1.26 (1.13-1.41)	<.001
**Educational**		
Illiterate	1.00 (Reference)	
Primary school and below	0.96 (0.83-1.11)	0.598
Secondary school	0.72 (0.60-0.86)	<.001
Junior college and above	0.47 (0.32-0.71)	<.001
**Marital**		
Married	1.00 (Reference)	
Unmarried	1.01 (0.89-1.14)	0.920
**Social security benefits received**		
None	1.00 (Reference)	
1	1.12 (0.82-1.53)	0.473
2	1.11 (0.74-1.66)	0.615
≥3	1.54 (0.94-2.52)	0.087
**Per capita disposable income**		
Did not exceed	1.00 (Reference)	
Exceeded	0.97 (0.73-1.28)	0.804
**Sleep**		
InSufficient	1.00 (Reference)	
sufficient	0.89 (0.77-1.03)	0.120
**BMI**		
Normal weight	1.00 (Reference)	
Thin	0.82 (0.64-1.05)	0.120
Overweight	1.11 (0.97-1.26)	0.141
Obese	1.90 (1.45-2.50)	<.001
**Physical activity**		
InActive	1.00 (Reference)	
active	0.55 (0.45-0.66)	<.001
**Number of medical and health institutions per 1000 people**		
Q1(The least)	1.00 (Reference)	
Q2	0.94 (0.61-1.45)	0.772
Q3	0.68 (0.43-1.09)	0.109
Q4(The most)	0.97 (0.61-1.53)	0.887
**Number of beds in medical and health institutions per 1000 people**		
Q1(The least)	1.00 (Reference)	
Q2	0.42 (0.27-0.64)	<.001
Q3	0.62 (0.40-0.96)	0.032
Q4(The most)	0.49 (0.31-0.77)	0.002

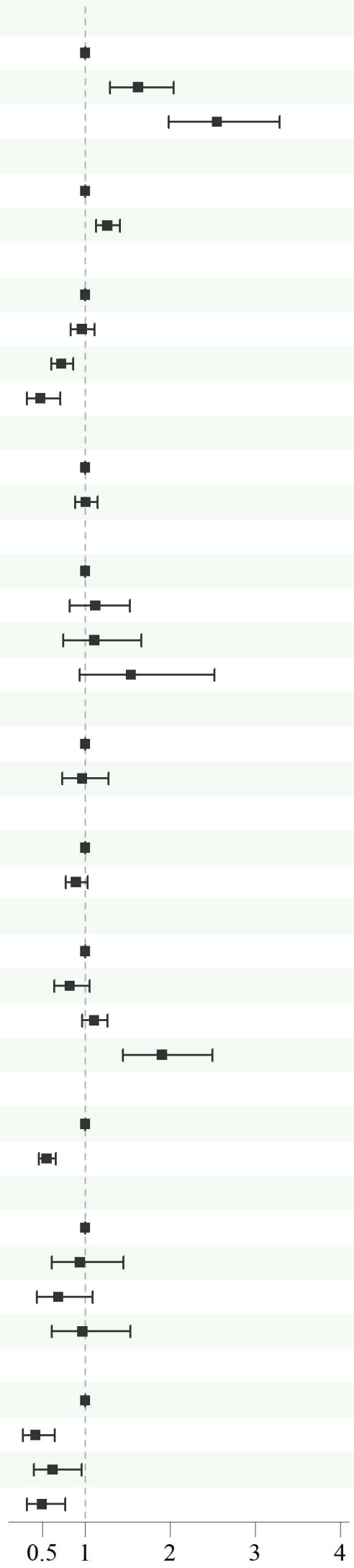

Supplementary Figure S6. Sensitivity analysis: Associations between socio-demographic/lifestyle variables and multimorbidity using an elevated diagnostic threshold (≥3 chronic conditions). The solid squares represent the Odds Ratios (OR), and the horizontal lines represent the 95% Confidence Intervals (CI) derived from the Generalized Linear Mixed Model. The vertical dashed line indicates an OR of 1.00. The corresponding P-values for each variable category are listed on the right.

Variables	OR (95% CI)	*P*-value
**Age (years)**		
60-68	1.00 (Reference)	
69-78	1.51 (1.26-1.81)	<.001
79-98	2.20 (1.79-2.71)	<.001
**Sex**		
Male	1.00 (Reference)	
Female	1.26 (1.16-1.38)	<.001
**Educational**		
Illiterate	1.00 (Reference)	
Primary school and below	0.84 (0.75-0.95)	0.007
Secondary school	0.64 (0.56-0.74)	<.001
Junior college and above	0.55 (0.40-0.75)	<.001
**Marital**		
Married	1.00 (Reference)	
Unmarried	1.05 (0.94-1.17)	0.367
**Social security benefits received**		
None	1.00 (Reference)	
1	1.15 (0.92-1.44)	0.228
2	1.32 (0.97-1.79)	0.078
≥3	1.57 (1.05-2.34)	0.029
**Per capita disposable income**		
Did not exceed	1.00 (Reference)	
Exceeded	0.94 (0.76-1.17)	0.574
**Sleep**		
InSufficient	1.00 (Reference)	
sufficient	0.92 (0.82-1.04)	0.203
**BMI**		
Normal weight	1.00 (Reference)	
Thin	0.88 (0.72-1.08)	0.219
Overweight	1.09 (0.98-1.22)	0.113
Obese	1.97 (1.55-2.52)	<.001
**Physical activity**		
InActive	1.00 (Reference)	
active	0.70 (0.61-0.82)	<.001
**Number of medical and health institutions per 1000 people**		
Q1(The least)	1.00 (Reference)	
Q2	1.02 (0.73-1.43)	0.915
Q3	0.63 (0.44-0.90)	0.011
Q4(The most)	0.77 (0.54-1.09)	0.142
**Number of beds in medical and health institutions per 1000 people**		
Q1(The least)	1.00 (Reference)	
Q2	0.48 (0.34-0.66)	<.001
Q3	0.75 (0.53-1.06)	0.102
Q4(The most)	0.59 (0.42-0.84)	0.003

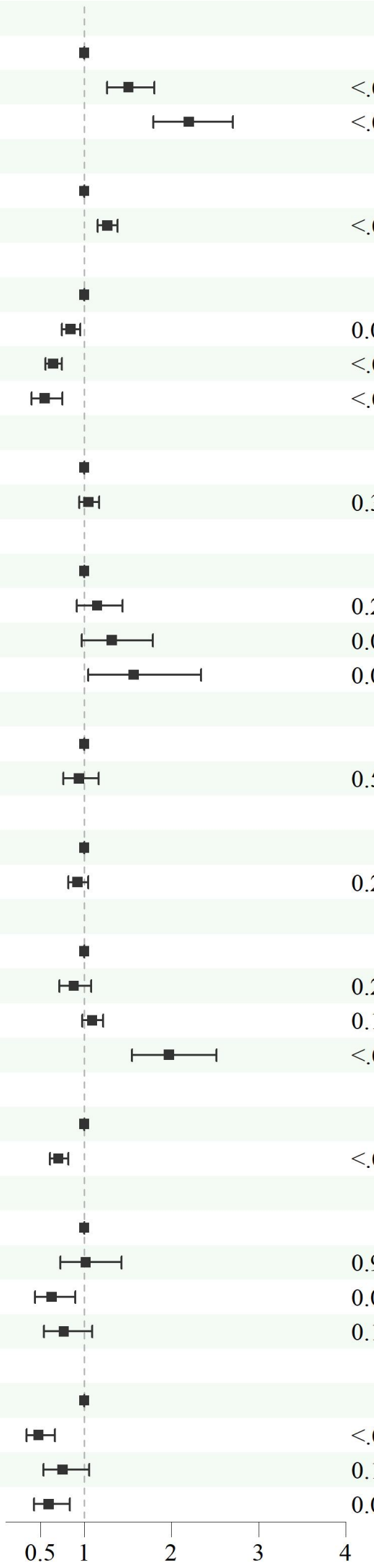

Supplementary Figure S7. Sensitivity analysis: Associations after excluding deafness and reproductive system diseases from the multimorbidity definition.

Variables	OR (95% CI)	*P*-value
**Age (years)**		
60-68	1.00 (Reference)	
69-78	1.35 (1.13-1.62)	0.001
79-98	2.10 (1.70-2.59)	<.001
**Sex**		
Male	1.00 (Reference)	
Female	1.27 (1.16-1.39)	<.001
**Educational**		
Illiterate	1.00 (Reference)	
Primary school and below	0.87 (0.77-0.99)	0.034
Secondary school	0.66 (0.57-0.76)	<.001
Junior college and above	0.56 (0.41-0.77)	<.001
**Marital**		
Married	1.00 (Reference)	
Unmarried	1.06 (0.95-1.19)	0.269
**Social security benefits received**		
None	1.00 (Reference)	
1	1.18 (0.94-1.49)	0.147
2	1.53 (1.12-2.08)	0.007
≥3	1.60 (1.07-2.41)	0.023
**Per capita disposable income**		
Did not exceed	1.00 (Reference)	
Exceeded	0.93 (0.74-1.16)	0.524
**Sleep**		
InSufficient	1.00 (Reference)	
sufficient	0.93 (0.82-1.06)	0.272
**BMI**		
Normal weight	1.00 (Reference)	
Thin	0.87 (0.71-1.06)	0.167
Overweight	1.10 (0.99-1.23)	0.090
Obese	1.95 (1.51-2.51)	<.001
**Physical activity**		
InActive	1.00 (Reference)	
active	0.72 (0.61-0.84)	<.001
**Number of medical and health institutions per 1000 people**		
Q1(The least)	1.00 (Reference)	
Q2	0.97 (0.68-1.40)	0.876
Q3	0.59 (0.40-0.87)	0.008
Q4(The most)	0.66 (0.45-0.96)	0.030
**Number of beds in medical and health institutions per 1000 people**		
Q1(The least)	1.00 (Reference)	
Q2	0.49 (0.35-0.71)	<.001
Q3	0.77 (0.53-1.11)	0.162
Q4(The most)	0.58 (0.40-0.83)	0.003

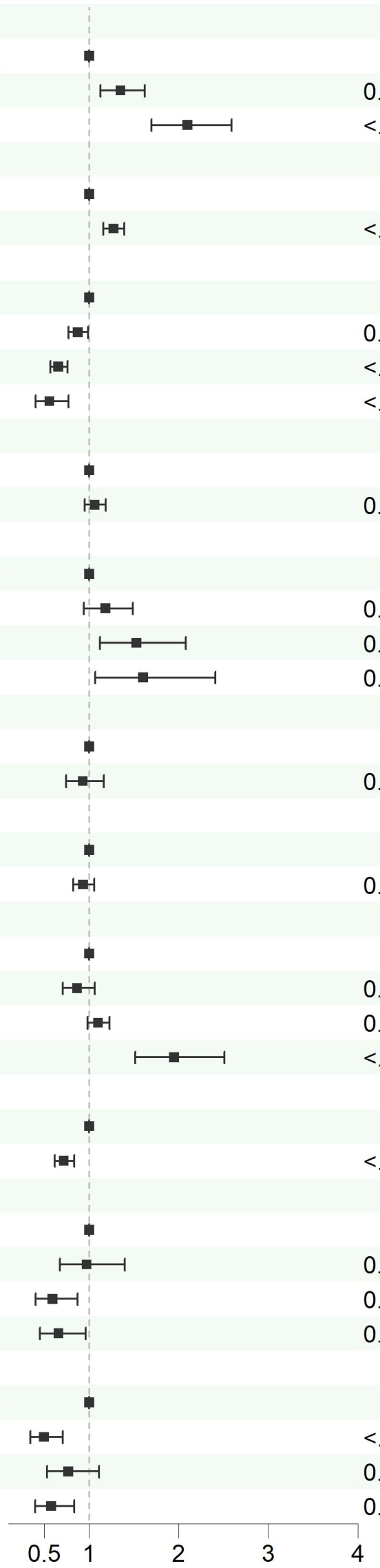

Supplementary Figure S8. Sensitivity analysis: Associations between socio-demographic, lifestyle and medical resource variables and multimorbidity after excluding participants with cognitive impairment-related conditions.
